# Supplementary material for: Modeling glioblastoma heterogeneity as a dynamic network of cell states
Source: Mol Syst Biol. 2021 Sep 16;17(9):e10105. doi: 10.15252/msb.202010105 (PMC8444284; doi:10.15252/msb.202010105)
Supplement: Supplementary file 6 — Source Data for Figure 5 [file MSB-17-e10105-s004.zip › Figure5A_sourcedata/GSEA_3071/nosigdb_stateIII.GseaPreranked.1622795155562/RICHARDS_NOWAKOWSKI_SCIENCE_2017_CHOROID_UPREG.html]

Details for gene set RICHARDS\_NOWAKOWSKI\_SCIENCE\_2017\_CHOROID\_UPREG[GSEA]

|  || Dataset | state33071 |
| Phenotype | NoPhenotypeAvailable |
| Upregulated in class | na\_pos |
| GeneSet | RICHARDS\_NOWAKOWSKI\_SCIENCE\_2017\_CHOROID\_UPREG |
| Enrichment Score (ES) | 0.23594563 |
| Normalized Enrichment Score (NES) | NaN |
| Nominal p-value | NaN |
| FDR q-value | 1.0 |
| FWER p-Value | 0.0 |
Table: GSEA Results Summary

  

Fig 1: Enrichment plot: RICHARDS\_NOWAKOWSKI\_SCIENCE\_2017\_CHOROID\_UPREG      
 Profile of the Running ES Score & Positions of GeneSet Members on the Rank Ordered List

  

| PROBE | GENE SYMBOL | GENE\_TITLE | RANK IN GENE LIST | RANK METRIC SCORE | RUNNING ES | CORE ENRICHMENT || 1 | HSPB1 |  |  | 4 | 0.650 | 0.0061 | Yes |
| 2 | RPLP0 |  |  | 6 | 0.633 | 0.0128 | Yes |
| 3 | RPS12 |  |  | 8 | 0.615 | 0.0194 | Yes |
| 4 | UQCRQ |  |  | 13 | 0.574 | 0.0246 | Yes |
| 5 | RPL35 |  |  | 17 | 0.549 | 0.0299 | Yes |
| 6 | RAB13 |  |  | 18 | 0.539 | 0.0358 | Yes |
| 7 | RPLP1 |  |  | 25 | 0.514 | 0.0399 | Yes |
| 8 | EEF1B2 |  |  | 28 | 0.501 | 0.0449 | Yes |
| 9 | SNRPD2 |  |  | 29 | 0.500 | 0.0504 | Yes |
| 10 | MT2A |  |  | 31 | 0.499 | 0.0557 | Yes |
| 11 | POLR2L |  |  | 38 | 0.482 | 0.0594 | Yes |
| 12 | RPS6 |  |  | 41 | 0.480 | 0.0641 | Yes |
| 13 | RPL8 |  |  | 44 | 0.469 | 0.0688 | Yes |
| 14 | RPL13A |  |  | 45 | 0.467 | 0.0740 | Yes |
| 15 | RPS20 |  |  | 49 | 0.463 | 0.0783 | Yes |
| 16 | NDUFB2 |  |  | 52 | 0.460 | 0.0828 | Yes |
| 17 | RPL32 |  |  | 55 | 0.457 | 0.0873 | Yes |
| 18 | RPL18A |  |  | 58 | 0.453 | 0.0918 | Yes |
| 19 | RPS18 |  |  | 61 | 0.451 | 0.0962 | Yes |
| 20 | COX8A |  |  | 63 | 0.448 | 0.1009 | Yes |
| 21 | RPS7 |  |  | 75 | 0.436 | 0.1028 | Yes |
| 22 | RPS3A |  |  | 82 | 0.429 | 0.1059 | Yes |
| 23 | OST4 |  |  | 83 | 0.429 | 0.1106 | Yes |
| 24 | MRPL33 |  |  | 87 | 0.423 | 0.1145 | Yes |
| 25 | UQCR10 |  |  | 89 | 0.421 | 0.1189 | Yes |
| 26 | RPS3 |  |  | 90 | 0.420 | 0.1235 | Yes |
| 27 | RPL10A |  |  | 94 | 0.414 | 0.1273 | Yes |
| 28 | RPS27A |  |  | 95 | 0.413 | 0.1319 | Yes |
| 29 | RPL35A |  |  | 96 | 0.413 | 0.1364 | Yes |
| 30 | S100A10 |  |  | 102 | 0.409 | 0.1396 | Yes |
| 31 | SERF2 |  |  | 111 | 0.403 | 0.1419 | Yes |
| 32 | UQCRB |  |  | 117 | 0.398 | 0.1450 | Yes |
| 33 | RPS23 |  |  | 129 | 0.391 | 0.1463 | Yes |
| 34 | NDUFA4 |  |  | 140 | 0.382 | 0.1478 | Yes |
| 35 | RPL13 |  |  | 152 | 0.372 | 0.1490 | Yes |
| 36 | CACYBP |  |  | 165 | 0.364 | 0.1498 | Yes |
| 37 | NEDD8 |  |  | 168 | 0.362 | 0.1532 | Yes |
| 38 | COX7C |  |  | 170 | 0.362 | 0.1570 | Yes |
| 39 | RPS15 |  |  | 180 | 0.353 | 0.1584 | Yes |
| 40 | FTH1 |  |  | 181 | 0.352 | 0.1623 | Yes |
| 41 | UBL5 |  |  | 182 | 0.352 | 0.1662 | Yes |
| 42 | SPATS2L |  |  | 184 | 0.351 | 0.1698 | Yes |
| 43 | RPS27L |  |  | 188 | 0.348 | 0.1729 | Yes |
| 44 | CHCHD2 |  |  | 195 | 0.341 | 0.1750 | Yes |
| 45 | PFDN2 |  |  | 202 | 0.338 | 0.1771 | Yes |
| 46 | LINC00467 |  |  | 206 | 0.335 | 0.1800 | Yes |
| 47 | GSTP1 |  |  | 211 | 0.333 | 0.1826 | Yes |
| 48 | COX4I1 |  |  | 223 | 0.326 | 0.1833 | Yes |
| 49 | NUDC |  |  | 226 | 0.325 | 0.1863 | Yes |
| 50 | ST13 |  |  | 227 | 0.325 | 0.1899 | Yes |
| 51 | RPS2 |  |  | 228 | 0.325 | 0.1935 | Yes |
| 52 | UQCC2 |  |  | 234 | 0.320 | 0.1957 | Yes |
| 53 | NDUFA2 |  |  | 239 | 0.315 | 0.1981 | Yes |
| 54 | ENO1 |  |  | 241 | 0.314 | 0.2013 | Yes |
| 55 | MZT2B |  |  | 244 | 0.313 | 0.2042 | Yes |
| 56 | SRP14 |  |  | 247 | 0.311 | 0.2071 | Yes |
| 57 | RPL23 |  |  | 250 | 0.310 | 0.2100 | Yes |
| 58 | OAZ1 |  |  | 259 | 0.305 | 0.2112 | Yes |
| 59 | NDUFB9 |  |  | 278 | 0.297 | 0.2097 | Yes |
| 60 | HSPE1 |  |  | 279 | 0.297 | 0.2130 | Yes |
| 61 | COX5B |  |  | 280 | 0.297 | 0.2162 | Yes |
| 62 | UBB |  |  | 294 | 0.293 | 0.2160 | Yes |
| 63 | TMEM258 |  |  | 295 | 0.292 | 0.2192 | Yes |
| 64 | PFDN5 |  |  | 302 | 0.290 | 0.2208 | Yes |
| 65 | PSME2 |  |  | 310 | 0.289 | 0.2221 | Yes |
| 66 | POLR2I |  |  | 311 | 0.289 | 0.2253 | Yes |
| 67 | NDUFB3 |  |  | 312 | 0.288 | 0.2285 | Yes |
| 68 | SOD1 |  |  | 315 | 0.287 | 0.2311 | Yes |
| 69 | MINOS1 |  |  | 321 | 0.286 | 0.2329 | Yes |
| 70 | TPT1 |  |  | 324 | 0.285 | 0.2355 | Yes |
| 71 | NAA38 |  |  | 354 | 0.274 | 0.2308 | Yes |
| 72 | RPL15 |  |  | 363 | 0.271 | 0.2316 | Yes |
| 73 | C11orf58 |  |  | 365 | 0.271 | 0.2343 | Yes |
| 74 | NDUFA11 |  |  | 371 | 0.269 | 0.2359 | Yes |
| 75 | MRFAP1 |  |  | 404 | 0.259 | 0.2302 | No |
| 76 | NDUFB8 |  |  | 428 | 0.251 | 0.2267 | No |
| 77 | ALDOA |  |  | 440 | -0.251 | 0.2266 | No |
| 78 | MNS1 |  |  | 463 | -0.252 | 0.2234 | No |
| 79 | ANKRD37 |  |  | 476 | -0.253 | 0.2230 | No |
| 80 | BCAP31 |  |  | 542 | -0.256 | 0.2083 | No |
| 81 | WDR66 |  |  | 584 | -0.259 | 0.2001 | No |
| 82 | ZFP36L2 |  |  | 671 | -0.263 | 0.1798 | No |
| 83 | CLDN1 |  |  | 692 | -0.264 | 0.1774 | No |
| 84 | TCP11L2 |  |  | 705 | -0.265 | 0.1770 | No |
| 85 | PRDM5 |  |  | 722 | -0.266 | 0.1757 | No |
| 86 | CRABP2 |  |  | 739 | -0.266 | 0.1743 | No |
| 87 | UNC50 |  |  | 763 | -0.267 | 0.1711 | No |
| 88 | GNS |  |  | 769 | -0.268 | 0.1727 | No |
| 89 | PBXIP1 |  |  | 771 | -0.268 | 0.1754 | No |
| 90 | CCDC181 |  |  | 797 | -0.269 | 0.1716 | No |
| 91 | PIP5K1B |  |  | 811 | -0.270 | 0.1711 | No |
| 92 | CAPS |  |  | 839 | -0.271 | 0.1668 | No |
| 93 | MSI2 |  |  | 848 | -0.272 | 0.1676 | No |
| 94 | IQCK |  |  | 869 | -0.272 | 0.1653 | No |
| 95 | ID3 |  |  | 880 | -0.273 | 0.1656 | No |
| 96 | FUCA2 |  |  | 884 | -0.274 | 0.1678 | No |
| 97 | SPAG1 |  |  | 893 | -0.274 | 0.1687 | No |
| 98 | FAF2 |  |  | 907 | -0.274 | 0.1682 | No |
| 99 | C21orf58 |  |  | 946 | -0.276 | 0.1610 | No |
| 100 | TMEM59 |  |  | 1010 | -0.280 | 0.1471 | No |
| 101 | NUDT7 |  |  | 1013 | -0.280 | 0.1497 | No |
| 102 | SULT1C4 |  |  | 1050 | -0.282 | 0.1431 | No |
| 103 | PRKCSH |  |  | 1118 | -0.286 | 0.1282 | No |
| 104 | LAMC1 |  |  | 1120 | -0.286 | 0.1311 | No |
| 105 | TMEM104 |  |  | 1130 | -0.286 | 0.1318 | No |
| 106 | SLC39A11 |  |  | 1182 | -0.289 | 0.1213 | No |
| 107 | IQCG |  |  | 1239 | -0.292 | 0.1094 | No |
| 108 | FSTL1 |  |  | 1254 | -0.293 | 0.1089 | No |
| 109 | IPO13 |  |  | 1272 | -0.293 | 0.1075 | No |
| 110 | PLEKHH2 |  |  | 1282 | -0.294 | 0.1083 | No |
| 111 | COL18A1 |  |  | 1302 | -0.295 | 0.1065 | No |
| 112 | PRNP |  |  | 1309 | -0.295 | 0.1081 | No |
| 113 | ATP2B4 |  |  | 1347 | -0.297 | 0.1014 | No |
| 114 | SLIT2 |  |  | 1400 | -0.300 | 0.0907 | No |
| 115 | FRMD8 |  |  | 1452 | -0.303 | 0.0803 | No |
| 116 | CADPS2 |  |  | 1505 | -0.306 | 0.0697 | No |
| 117 | PCCA |  |  | 1508 | -0.306 | 0.0726 | No |
| 118 | WLS |  |  | 1517 | -0.307 | 0.0738 | No |
| 119 | IFT122 |  |  | 1539 | -0.308 | 0.0715 | No |
| 120 | ARHGAP29 |  |  | 1614 | -0.313 | 0.0551 | No |
| 121 | GTF2E1 |  |  | 1629 | -0.314 | 0.0548 | No |
| 122 | GK |  |  | 1637 | -0.315 | 0.0563 | No |
| 123 | PLK4 |  |  | 1642 | -0.315 | 0.0587 | No |
| 124 | KIF9 |  |  | 1646 | -0.315 | 0.0614 | No |
| 125 | SPICE1 |  |  | 1658 | -0.316 | 0.0619 | No |
| 126 | PLEKHA4 |  |  | 1672 | -0.317 | 0.0619 | No |
| 127 | MSX1 |  |  | 1715 | -0.320 | 0.0542 | No |
| 128 | PEG3 |  |  | 1754 | -0.322 | 0.0475 | No |
| 129 | CLMN |  |  | 1773 | -0.323 | 0.0462 | No |
| 130 | NUDT6 |  |  | 1813 | -0.325 | 0.0393 | No |
| 131 | ABLIM1 |  |  | 1940 | -0.332 | 0.0090 | No |
| 132 | EFHC1 |  |  | 1946 | -0.332 | 0.0113 | No |
| 133 | FAH |  |  | 2007 | -0.335 | -0.0012 | No |
| 134 | ISYNA1 |  |  | 2010 | -0.336 | 0.0020 | No |
| 135 | TIMP1 |  |  | 2038 | -0.337 | -0.0015 | No |
| 136 | RDH10 |  |  | 2051 | -0.338 | -0.0010 | No |
| 137 | TANC1 |  |  | 2069 | -0.339 | -0.0019 | No |
| 138 | PPIB |  |  | 2111 | -0.342 | -0.0091 | No |
| 139 | IFT46 |  |  | 2140 | -0.344 | -0.0129 | No |
| 140 | USP51 |  |  | 2201 | -0.349 | -0.0252 | No |
| 141 | CGNL1 |  |  | 2224 | -0.350 | -0.0273 | No |
| 142 | SASH1 |  |  | 2231 | -0.351 | -0.0250 | No |
| 143 | SERPINF1 |  |  | 2245 | -0.352 | -0.0246 | No |
| 144 | BTD |  |  | 2250 | -0.353 | -0.0218 | No |
| 145 | CLHC1 |  |  | 2284 | -0.355 | -0.0268 | No |
| 146 | BSG |  |  | 2316 | -0.359 | -0.0311 | No |
| 147 | GJA1 |  |  | 2362 | -0.362 | -0.0393 | No |
| 148 | CYB561 |  |  | 2441 | -0.368 | -0.0562 | No |
| 149 | SGMS2 |  |  | 2450 | -0.369 | -0.0543 | No |
| 150 | GALNT5 |  |  | 2488 | -0.372 | -0.0602 | No |
| 151 | PRCP |  |  | 2489 | -0.372 | -0.0560 | No |
| 152 | PDIA6 |  |  | 2505 | -0.373 | -0.0560 | No |
| 153 | LAMB1 |  |  | 2556 | -0.378 | -0.0653 | No |
| 154 | STK33 |  |  | 2562 | -0.379 | -0.0624 | No |
| 155 | ADAMTS6 |  |  | 2604 | -0.382 | -0.0692 | No |
| 156 | DHRS1 |  |  | 2634 | -0.384 | -0.0728 | No |
| 157 | MDM1 |  |  | 2643 | -0.385 | -0.0707 | No |
| 158 | CKAP4 |  |  | 2664 | -0.387 | -0.0718 | No |
| 159 | SLC2A10 |  |  | 2676 | -0.388 | -0.0705 | No |
| 160 | ID1 |  |  | 2685 | -0.389 | -0.0684 | No |
| 161 | LMAN1 |  |  | 2743 | -0.394 | -0.0794 | No |
| 162 | COBLL1 |  |  | 2791 | -0.398 | -0.0876 | No |
| 163 | ITGA6 |  |  | 2899 | -0.408 | -0.1120 | No |
| 164 | SERPINB1 |  |  | 2918 | -0.410 | -0.1123 | No |
| 165 | DSG2 |  |  | 2922 | -0.411 | -0.1085 | No |
| 166 | WDR83 |  |  | 2958 | -0.415 | -0.1134 | No |
| 167 | GCHFR |  |  | 2963 | -0.415 | -0.1099 | No |
| 168 | MITF |  |  | 2968 | -0.416 | -0.1064 | No |
| 169 | PPIC |  |  | 3045 | -0.425 | -0.1222 | No |
| 170 | LEPR |  |  | 3057 | -0.426 | -0.1204 | No |
| 171 | TCF7 |  |  | 3070 | -0.428 | -0.1189 | No |
| 172 | USP54 |  |  | 3086 | -0.429 | -0.1182 | No |
| 173 | RAB3IP |  |  | 3101 | -0.431 | -0.1172 | No |
| 174 | HSPA5 |  |  | 3193 | -0.442 | -0.1369 | No |
| 175 | MYO10 |  |  | 3196 | -0.442 | -0.1325 | No |
| 176 | SLC5A6 |  |  | 3204 | -0.443 | -0.1295 | No |
| 177 | SEMA5A |  |  | 3229 | -0.447 | -0.1310 | No |
| 178 | SLC29A4 |  |  | 3244 | -0.450 | -0.1298 | No |
| 179 | FZD1 |  |  | 3256 | -0.452 | -0.1278 | No |
| 180 | CD63 |  |  | 3258 | -0.452 | -0.1231 | No |
| 181 | P4HTM |  |  | 3275 | -0.455 | -0.1224 | No |
| 182 | CNKSR3 |  |  | 3281 | -0.456 | -0.1187 | No |
| 183 | ITM2B |  |  | 3314 | -0.463 | -0.1222 | No |
| 184 | MEST |  |  | 3363 | -0.473 | -0.1299 | No |
| 185 | AKNA |  |  | 3388 | -0.479 | -0.1311 | No |
| 186 | RAB11FIP1 |  |  | 3394 | -0.480 | -0.1271 | No |
| 187 | PLBD1 |  |  | 3411 | -0.483 | -0.1261 | No |
| 188 | JAG1 |  |  | 3424 | -0.486 | -0.1239 | No |
| 189 | FAM210B |  |  | 3447 | -0.492 | -0.1244 | No |
| 190 | TMEM67 |  |  | 3465 | -0.497 | -0.1235 | No |
| 191 | COL21A1 |  |  | 3488 | -0.504 | -0.1239 | No |
| 192 | LRRC8C |  |  | 3500 | -0.507 | -0.1212 | No |
| 193 | CACNB2 |  |  | 3564 | -0.532 | -0.1323 | No |
| 194 | ILDR2 |  |  | 3592 | -0.544 | -0.1336 | No |
| 195 | ARSA |  |  | 3604 | -0.551 | -0.1305 | No |
| 196 | IGFBP7 |  |  | 3611 | -0.554 | -0.1260 | No |
| 197 | ANLN |  |  | 3625 | -0.562 | -0.1233 | No |
| 198 | TPBG |  |  | 3638 | -0.570 | -0.1202 | No |
| 199 | SLC5A3 |  |  | 3647 | -0.575 | -0.1160 | No |
| 200 | AHNAK |  |  | 3660 | -0.584 | -0.1128 | No |
| 201 | PDPN |  |  | 3676 | -0.597 | -0.1102 | No |
| 202 | FAT1 |  |  | 3685 | -0.604 | -0.1057 | No |
| 203 | APLP2 |  |  | 3693 | -0.610 | -0.1009 | No |
| 204 | BMP7 |  |  | 3698 | -0.613 | -0.0952 | No |
| 205 | COLEC12 |  |  | 3699 | -0.613 | -0.0884 | No |
| 206 | SYT17 |  |  | 3701 | -0.614 | -0.0819 | No |
| 207 | ENPP2 |  |  | 3713 | -0.627 | -0.0779 | No |
| 208 | FAM84A |  |  | 3729 | -0.639 | -0.0749 | No |
| 209 | STON2 |  |  | 3739 | -0.658 | -0.0700 | No |
| 210 | HSP90B1 |  |  | 3757 | -0.681 | -0.0671 | No |
| 211 | SOSTDC1 |  |  | 3773 | -0.705 | -0.0633 | No |
| 212 | EFEMP1 |  |  | 3784 | -0.716 | -0.0581 | No |
| 213 | FBN1 |  |  | 3790 | -0.723 | -0.0515 | No |
| 214 | ELN |  |  | 3818 | -0.766 | -0.0503 | No |
| 215 | CLU |  |  | 3846 | -0.827 | -0.0484 | No |
| 216 | PROM1 |  |  | 3854 | -0.846 | -0.0410 | No |
| 217 | CXCL14 |  |  | 3892 | -1.060 | -0.0392 | No |
| 218 | CTGF |  |  | 3901 | -1.185 | -0.0283 | No |
| 219 | COL3A1 |  |  | 3918 | -1.418 | -0.0169 | No |
| 220 | PDGFRA |  |  | 3928 | -1.773 | 0.0003 | No |
Table: GSEA details [plain text format]

  

Fig 2: RICHARDS\_NOWAKOWSKI\_SCIENCE\_2017\_CHOROID\_UPREG: Random ES distribution      
 Gene set null distribution of ES for **RICHARDS\_NOWAKOWSKI\_SCIENCE\_2017\_CHOROID\_UPREG**
